# Cancrum Oris

**Published:** 1887-01

**Authors:** 


ARTICLE VIII.

### CANCRUM ORIS.

At a meeting of the Cambridge Medical Society, held on October 8th, Mr. Wherry related a case of cancrum oris that was lately under his care in Addenbrooke's Hospital. A female domestic servant, aged twenty-five years, had a deep ulcer filled with yellow slough, occupying the whole of the inside of the left cheek, said to have begun as a gum-boil. The ulcer was almost painless. The skin of the left cheek was dusky red in color over the slough, was nearly destroyed, and eventually there was a perforation through the cheek. The separation of the slough was at no time accompanied by any hæmorrhage, there seemed to be no granulating surface to bleed, and there was only a thin watery discharge and faintly tinged saliva. Her complexion was remarkably pale-yellow and wax-like. The fever was high and the pulse rapid. The register on the chart was usually 104 degrees to 105 degrees, and the pulse 160 or more. She had been ailing four or five weeks, and had suffered from the ulcer two weeks before admission. No treatment was of any value in checking the progress of the malady, and she died exhausted in twelve days. She had always a good appetite, partook heartily of a liberal diet, and did not consider herself very ill. On two occasions operations were performed under ether. At first the slough was cleared out and the cavity left was swabbed freely with nitric acid. At a later stage the cheek was laid freely open from the outside. Quinine stopped the rigors which occurred occasionally. Iron was also given, and later on opium. Condy's fluid and insufflation of iodoform were tried locally. This patient was always pale and perhaps anæmic, but she was not ill-fed or ill-nourished, nor was she recovering from any acute fever. She had been able to do her duty as a domestic servant until attacked by disease. Mr. Wherry mentioned the reasons for making a difference between cancrum oris and other conditions somewhat similar, as ulcerative stomatitis. It was not a case of acute necrosis of the upper jaw. Though as yet no organism had been isolated from the blood or tissues, nor had the disease been inoculated, it seemed probable that in the future, as in the case of anthrax, a definite micro-organism would be discovered in cancrum oris. Animals injected with the blood of patients suffering from cancrum oris died of septicæmia, usually with peritonitis.—*The Dental Record.*

---

ARTICLE IX.

